# Osteomyelitis of Frontal Sinus: A Rare Sequelae of Acute Bacterial Sinusitis Associated With Anatomical Variations in the Frontal Sinus Drainage Pathway

**DOI:** 10.7759/cureus.54158

**Published:** 2024-02-13

**Authors:** Smriti Wadhwa, Shraddha Jain, Jaya Gupta, Harshil Dobariya, Nikhil Pantbalekundri

**Affiliations:** 1 Department of Otorhinolaryngology - Head and Neck Surgery, Jawaharlal Nehru Medical College, Datta Meghe Institute of Higher Education and Research (Deemed to be university), Wardha, IND; 2 Department of Medicine, Jawaharlal Nehru Medical College, Datta Meghe Institute of Higher Education and Research (Deemed to be university), Wardha, IND

**Keywords:** endoscopic sinus surgery, drainage pathway, sinusitis, frontal sinus, pansinusitis, osteomyelitis

## Abstract

Frontal osteomyelitis is characterized by localized osteal inflammation of the frontal bone. This is a rare complication of acute frontal sinusitis. The present case is being reported to highlight the likely role of anatomical variations in frontal sinus drainage pathways in the causation of this complication apart from other known predisposing factors like young age and immunocompromised state. The patient initially presented with seizures, fever, and headache and was diagnosed with viral encephalitis. However, the symptoms gradually progressed to cause right eye swelling and an increase in the severity of headache without any nasal complaints. Diagnostic nasal endoscopy revealed mucopurulent secretions in both nasal cavities. Computed tomography and magnetic resonance imaging diagnosed the anatomical variations, the extent of sinus involvement, and frontal osteomyelitis. Antimicrobial therapy for an extended duration of four weeks, along with functional endoscopic sinus surgery resulted in excellent outcomes.

## Introduction

Frontal osteomyelitis can develop as a result of frontal sinusitis or trauma, developing into frontal osteomyelitis with accompanying subperiosteal abscess. The classic triad of frontal sinusitis, osteomyelitis, and subperiosteal abscess formation is characteristic of Pott’s puffy tumor (PPT) [1]. It is predominant in the younger population with a male predominance. Frontal sinuses begin to pneumatize by two years of life and are nearly fully grown by puberty, favoring the development of osteomyelitis [2]. Infection travels from the sinus to the subgaleal space via the diploic veins, resulting in frontal bone osteomyelitis, erosion through the frontal bone, and subperiosteal abscess development. Posterior spread can result in extradural empyema, subdural empyema, an inflammatory reaction in the subarachnoid space, or brain parenchyma involvement [3]. Headache, purulent rhinorrhea, fever, frontal sinus pain, and periorbital edema are the most prevalent symptoms. Vomiting, headache, fever, change in mental status, lethargy, seizure, and other clinical symptoms may indicate intracranial problems [1,2].

The frontal sinus drainage pathway shows anatomical variations among individuals [4]. The drainage pathway is influenced by the anatomical variations of the middle turbinate, uncinate, and other cells. All this contributes to the complexity of the disease, influencing both its clinical presentation and diagnostic considerations. Here, we report a case of an adolescent male patient, who develops frontal osteomyelitis as a sequel of acute closed pansinusitis, likely precipitated due to anatomical variations in the frontal sinus drainage pathway.

## Case presentation

**Figure 1:**
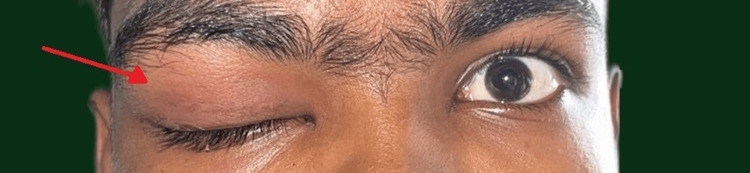
Right periorbital swelling (red arrow).

A 17-year-old boy presented to the Department of Otorhinolaryngology with complaints of right orbital swelling (Figure 1) and headache for the past three weeks, which was of a throbbing type, intermittent, moderate in intensity, lasting for one hour, hindered sleep and day-to-day activities, and was associated with fever. The patient also had a history of one episode of generalized seizure which was then followed by the above symptoms. The patient was being handled by a neurologist at another center and was diagnosed with viral encephalitis based on magnetic resonance imaging (MRI) findings. Due to the occurrence of orbital symptoms and the persistence of headache, the otorhinolaryngologist's opinion was sought. On Ophthalmologic consultation, all the parameters were within normal limits.

**Figure 2:**
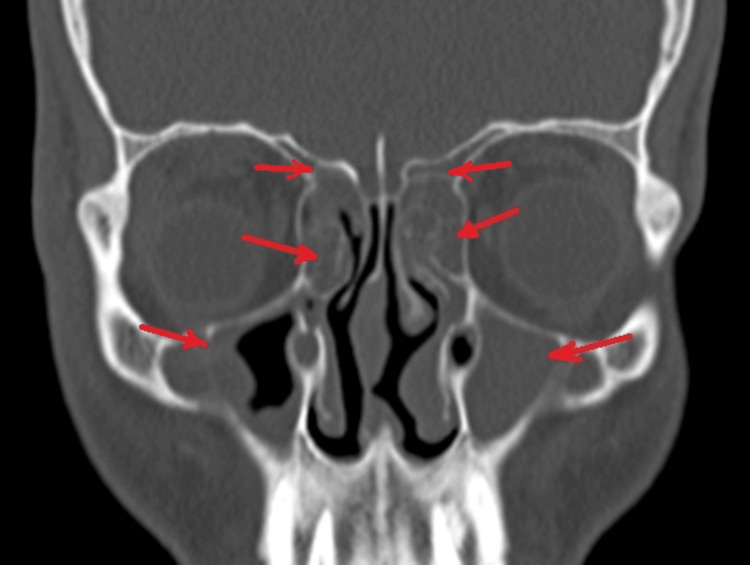
Contrast-enhanced CT showing collection in bilateral maxillary, ethmoidal, and frontal sinuses (red arrows).

**Figure 3:**
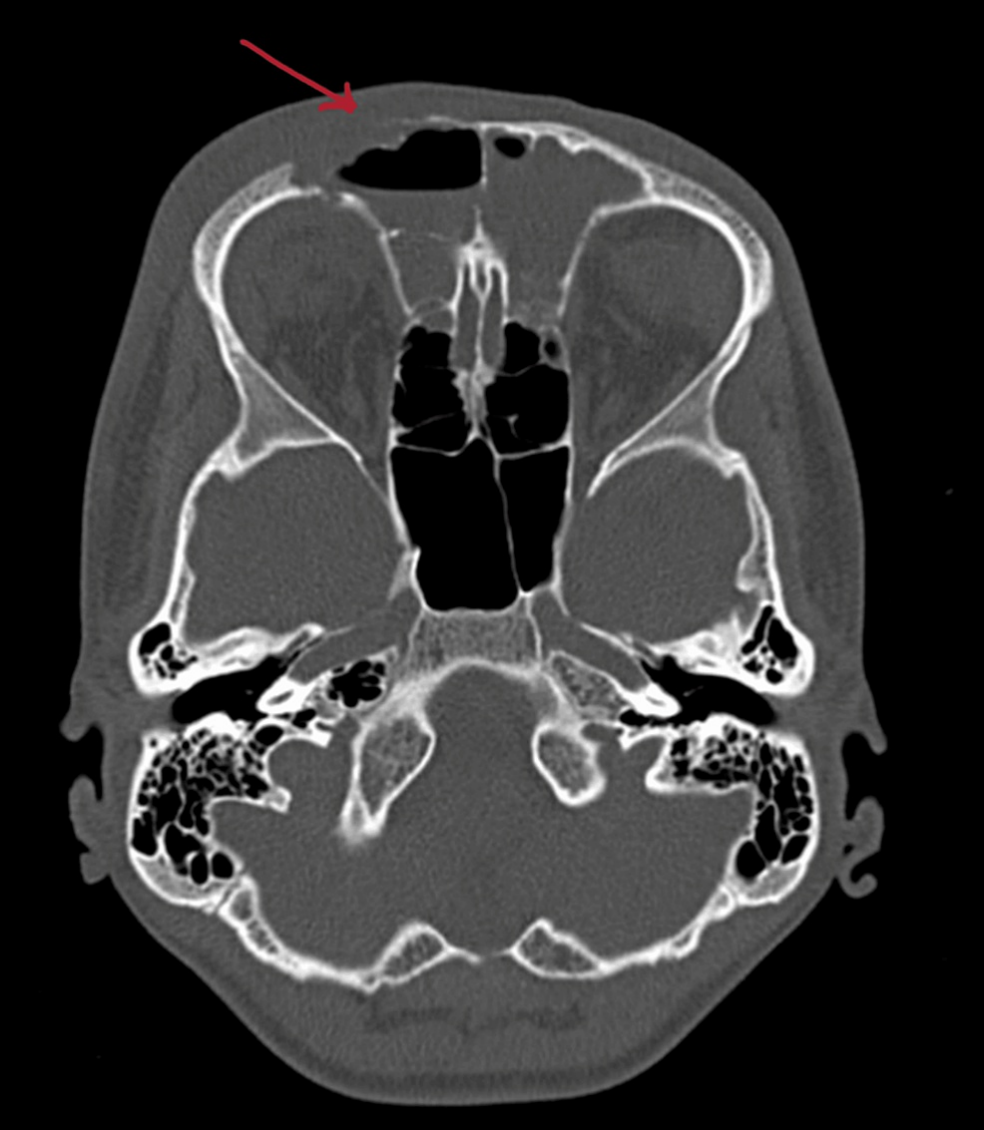
Erosion of the outer wall of the frontal sinus and the adjacent bony orbital roof (indicated with red arrow).

**Figure 4:**
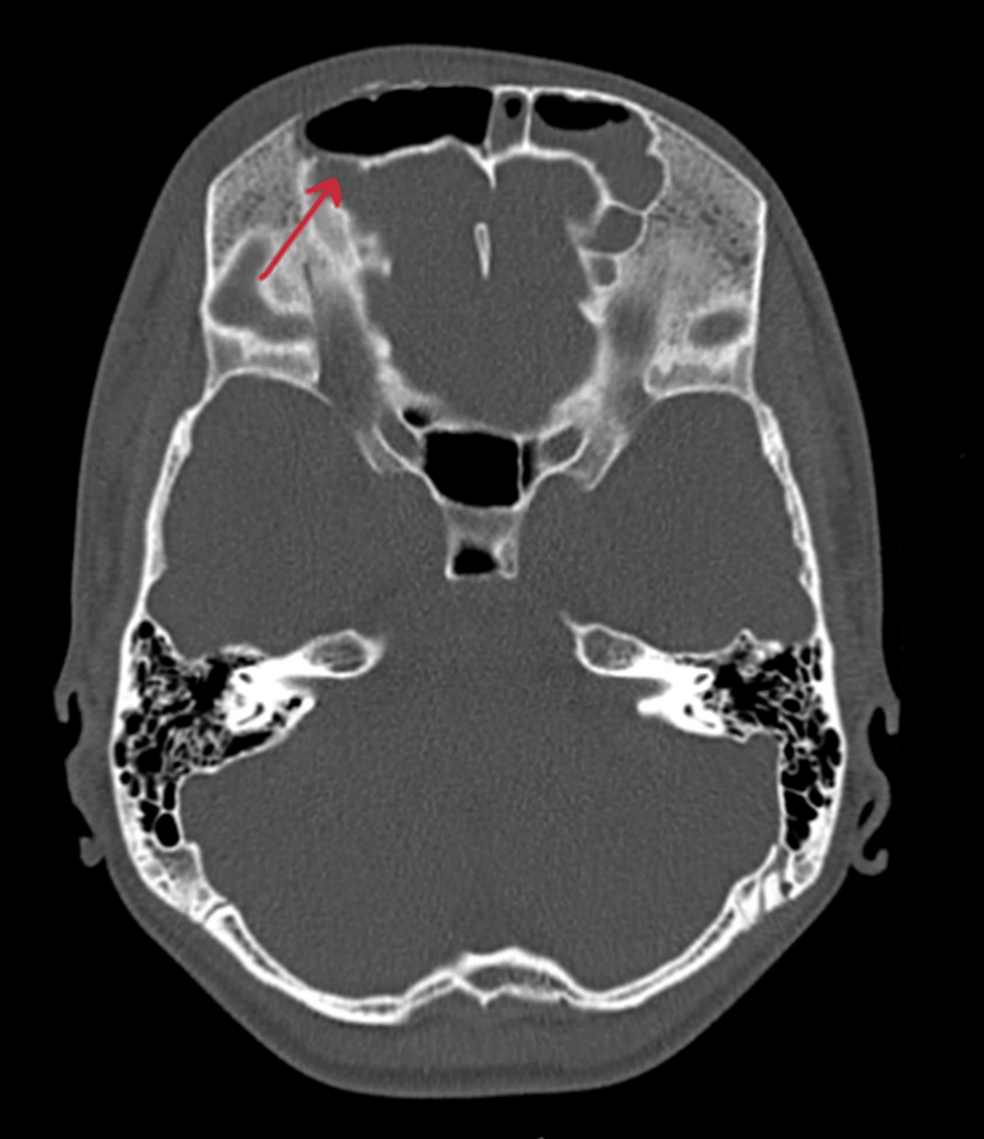
Contrast-enhanced CT showing erosion of the inner table of the frontal sinus (indicated with red arrow).

**Figure 5:**
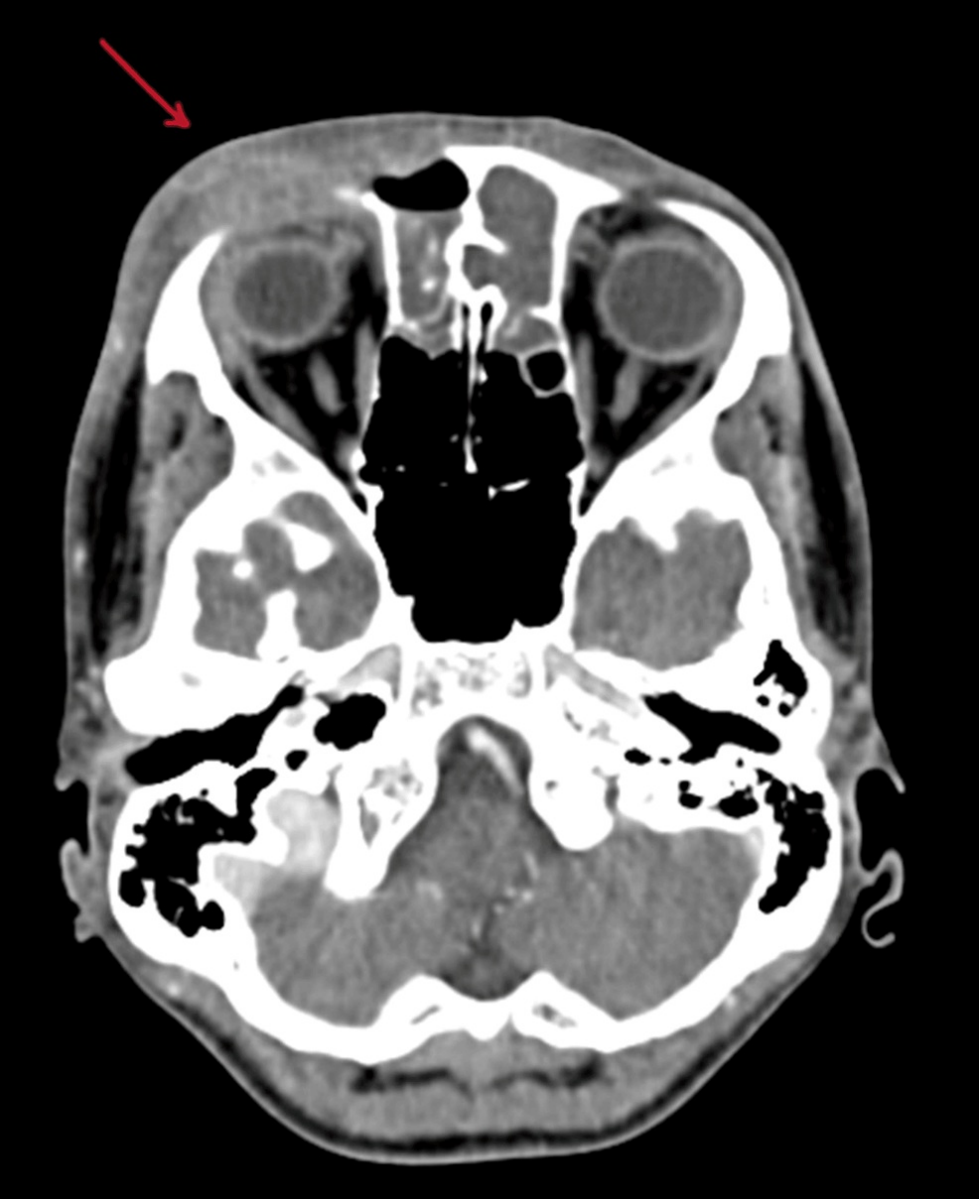
Contrast-enhanced CT showing periorbital cellulitis (indicated with red arrow).

On contrast-enhanced computed tomography (CECT) of paranasal sinuses, collection was reported in bilateral maxillary, anterior ethmoid, and frontal sinuses (Figure 2) with bony erosion of the outer (Figure 3) and inner table of the right frontal sinus (Figure 4) and roof of the right orbit, most likely suggestive of acute invasive fungal sinusitis or bacterial sinusitis with osteomyelitis. Also, there was soft tissue thickening/edema involving the right pre-septal space and right peri-orbital region suggesting peri-orbital cellulitis (Figure 5), drawing the possibility of infective etiology involving the superior wall of the right orbit and adjacent right frontal bone with mild extraosseous abnormal soft tissue seen involving the right frontal scalp. Diagnosis of right frontal osteomyelitis and reactive marrow edema, with pre-septal cellulitis as a complication of pansinusitis was made.

**Figure 6:**
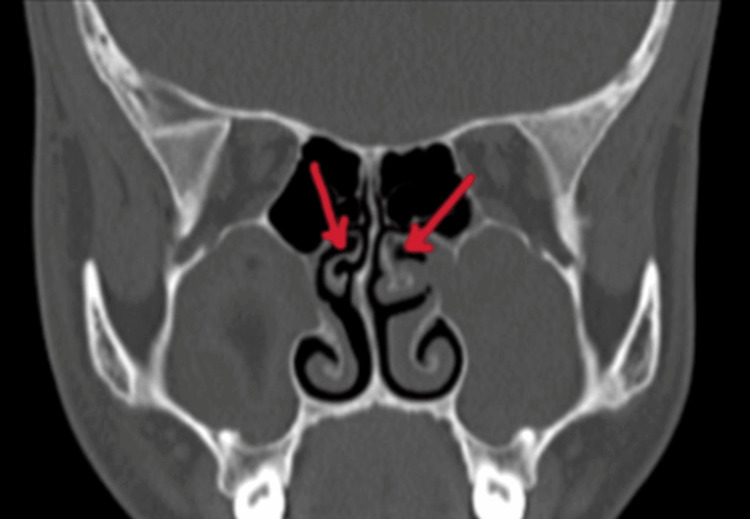
CT showing paradoxical curvature of middle turbinates (as indicated with red arrows).

**Figure 7:**
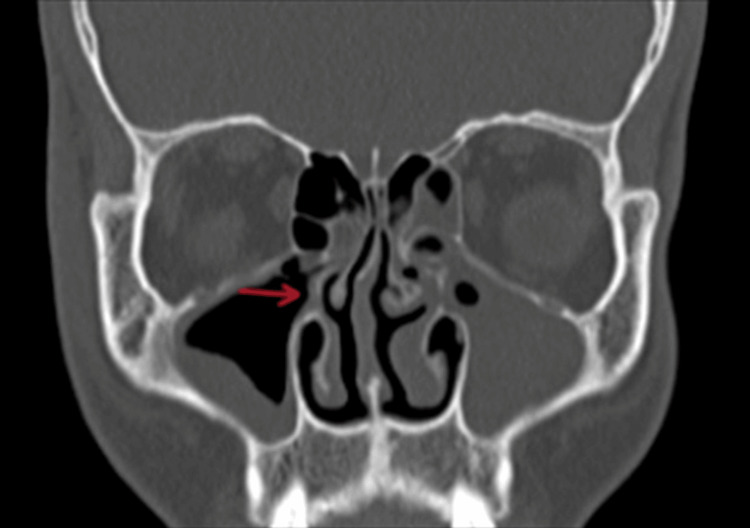
CT showing attachment of the right uncinate process to the middle turbinate (indicated by a red arrow).

On diagnostic nasal endoscopy, mucopurulent discharge was found in the bilateral middle meatus. There was no blackish discoloration of turbinate or the presence of fungal debris, suggesting a bacterial etiology. Bilateral middle turbinates were found to have paradoxical curvature (Figure 6). Uncinate was found to be attached to the middle turbinate and the frontal sinus pathway was lateral as confirmed on CECT, paranasal sinuses (Figure 7). The patient was then posted for functional endoscopic sinus surgery (FESS). Bilateral middle turbinectomy with uncinectomy was done.

**Figure 8:**
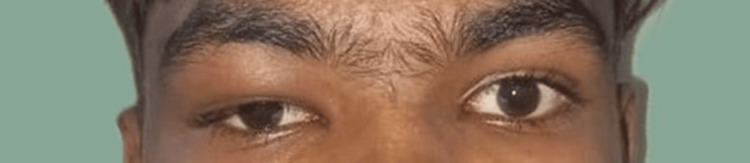
Patient in the immediate post-operative period with significantly reduced periorbital swelling.

Mucopurulent discharge was seen draining from both maxillary and frontal sinus ostia. Therefore, both maxillary sinuses were widened, and anterior ethmoid sinuses and frontal sinuses were opened. Intravenous antibiotics comprising a combination of piperacillin and tazobactam, vancomycin, linezolid, and clindamycin were given for two weeks followed by oral antibiotics for two weeks on discharge (Figure 8). There was full recovery (clinically and symptomatically) at the one-month follow-up and the patient had no complaints at the three-month follow-up.

## Discussion

Upper respiratory tract infection, head trauma, or local nasal factors leading to blockage of osteomeatal complex (OMC) are some factors that are found to precede the development of acute frontal sinusitis [5]. OMC represents the common pathway for drainage and ventilation of the frontal, maxillary, and anterior ethmoid air cells. The identification of the drainage pathway is critical preoperatively. The obstruction of this region can lead to the development of chronic sinusitis [6]. Narrowness in the OMC could be caused by anatomic differences in intrinsic structures (such as the uncinate process or ethmoid bulla) as well as extrinsic structures (such as middle turbinate hypertrophy, Haller cells, septal deviation, or a combination of these). The uncinate can be attached to lamina papyracea, skull base, or middle concha, and can have variations like bifid uncinate, uncinate bulla, a deviated tip of uncinate, curved uncinate, or hypoplastic/atelectatic [7]. Middle concha may present a curvature convexity laterally called paradoxical middle concha [8]. A prominent medial curve or bend of the uncinate process is the most common and pathologically relevant variant. One of the most common clinical features in patients with chronic rhinosinusitis is a significantly medially bent or folded uncinate process with a corresponding area of substantial contact with the middle turbinate [9]. In our patient, young age along with variations found in drainage pathways in the form of the paradoxical middle turbinate and attachment of uncinate to middle turbinate leading to lateral drainage of the frontal sinus might have led to nonresolution of symptoms with resultant bony and orbital complications.

Acute frontal sinusitis can lead to orbital or bony complications. It could be of bacterial or fungal etiology [10-12]. Frontal osteomyelitis has been reported following both acute and chronic bacterial frontal sinusitis [13]. The one following acute is more common in adolescents. Our patient was an adolescent male with a higher likelihood of frontal osteomyelitis due to diploic veins leading to anterior spread and intracranial complications due to posterior spread [3]. Up to 30% of cases of PPT are associated with intracranial complications, in the form of frontal sinusitis, osteomyelitis, and subperiosteal abscess. The existence of intracranial problems, neurological clinical indications, and worsening of common symptoms 48 hours after intravenous antibiotic therapy suggests that surgical drainage is required immediately [1]. There was no frank abscess formation either subperiosteal or intracranial; however, the patient had forehead scalp edema and pre-septal cellulitis. He also had an intracranial infection in the form of encephalitis, which resolved on medical treatment. The patient had frontal osteomyelitis and was at a stage of cellulitis, which could have progressed to frank PPT with abscess formation [12].

In the majority of PPT cases (those with gradual remission of symptoms and no intracranial problems), early treatment with IV broad-spectrum antibiotics can suffice, and simple drainage can be performed if necessary. When the patient enters the chronic phase with sequestrum formation, surgical intervention should be delayed and conducted in an inflammatory-free surgical area [4,13]. Surgical options are an open approach which includes osteoplastic flaps, Reidel procedures, and cranialization. With the advent of endoscopy, endoscopic approaches like Draf IIB, and Draf III can be undertaken when we intend to remove the devitalized frontal bone [14]. However, in our patient, addressing the drainage pathways by FESS and medical management gave excellent outcomes, as it was in the acute phase of osteomyelitis and had not progressed to the chronic phase [15].

The surgical decision-making about endoscopic (simple FESS/ Draf) versus open approaches should be based on the degree, extent, and localization of devitalized bone and the chronicity of the disease [14].

## Conclusions

The presented case of frontal osteomyelitis emphasizes the crucial role of recognizing and addressing anatomical variations within the frontal sinus drainage pathways, identification of the stage of the disease (acute/chronic), and the role of appropriate medical management. Occurrence of intracranial complications can present to a neurologist or neurosurgeon, who should be aware of the likelihood of frontal sinus as a source of infection. A high index of suspicion and early referral to an Otolaryngologist would lead to early appropriate diagnosis and timely management of the condition to avoid life-threatening complications.
